# Co-circulation of Crimean-Congo Hemorrhagic Fever virus strains Asia 1 and 2 between the border of Iran and Pakistan

**DOI:** 10.1016/j.heliyon.2017.e00439

**Published:** 2017-11-13

**Authors:** Nariman Shahhosseini, Ahmad Jafarbekloo, Zakkyeh Telmadarraiy, Sadegh Chinikar, Ali Haeri, Norbert Nowotny, Martin H. Groschup, Anthony R. Fooks, Faezeh Faghihi

**Affiliations:** a Bernhard Nocht Institute for Tropical Medicine, WHO Collaborating Centre for Arbovirus and Haemorrhagic Fever Reference and Research, Department of Virology, Hamburg, Germany; b Medical School, Tehran University of Medical Sciences, Tehran, Iran; c Department of Entomology, Tehran University of Medical Science, Tehran, Iran; d Pasteur Institute of Iran, Tehran, Iran; e Shahid Beheshti University of Medical Sciences, Tehran, Iran; f Institute of Virology, Department of Pathobiology, University of Veterinary Medicine, Vienna, Austria; g Department of Basic Medical Sciences, College of Medicine, Mohammed Bin Rashid University of Medicine and Health Sciences, Dubai Healthcare City, Dubai, United Arab Emirates; h Friedrich-Loeffler-Institut, Federal Research Institute for Animal Health, Greifswald-Insel Riems, Germany; i Wildlife Zoonoses and Vector-borne Diseases Research Group, Animal and Plant Health Agency, Woodham Lane, New Haw, Surrey, KT15 3NB, UK; j Department of Clinical Infection, Microbiology and Immunology, University of Liverpool, Liverpool, UK; k Cellular and Molecular Research Center, Iran University of Medical Sciences, Tehran, Iran

**Keywords:** Virology

## Abstract

Crimean-Congo Hemorrhagic Fever (CCHF) is a tick-borne viral disease that is transmitted by numerous species of ticks, which serve both as a reservoir and vector of CCHF virus (CCHFV). Molecular and serological tests were undertaken on hard ticks (Ixodidae spp.) and samples from livestock were collected in 2015 from Chabahar County in Southeast Iran. Using RT-PCR, the ticks were tested for the presence of CCHFV. In addition, seven livestock were serologically tested for the presence of IgG antibodies using an ELISA test. IgG antibodies against CCHFV were detected in one of 7 of the livestock that were tested. In total, 49 ticks including five species: *Rhipicephalus sanguineus, Hyalomma anatolicum*, *Hy. asiaticum, Hy. dromedarii* and *Hy. marginatum* with a prevalence of 46.9%, 32.7%, 4.1%, 4.1% and 2.1% respectively were identified. CCHFV was detected in three ticks among 49 collected ticks. The ticks infected with CCHFV belonged to the genus *Hyalomma* and *Rhipicephalus.* Phylogenetic analysis demonstrated that two sequences clustered in clade IV (Asia-1) and one sequence was located within clade IV (Asia-2). Most of the animal and human CCHF cases of the country are reported from Sistan and Baluchistan provinces. Regular monitoring programs in the tick population and livestock are needed in the future.

## 1 Introduction

Crimean-Congo hemorrhagic fever (CCHF) is an acute, viral, zoonotic disease with hemorrhagic manifestations and a considerable mortality rate in humans [1]. The disease has a worldwide distribution and is considered as an endemic disease in many countries of Asia, Europe, and Africa. New outbreaks of this disease were recorded in Kosovo, Senegal, Turkey, Bulgaria, Iran, Pakistan and Mauritania [2, 3].

CCHFV is a single stranded RNA virus, *Nairovirus* genus from the Bunyaviridae family, with a segmented negative sense genome consisting of a small (S), a medium (M), and a large (L) segment. CCHFVs are relatively divergent in their genome sequence and grouped into seven distinct clades based on the S-segment genome analysis: West-Africa in clade I, Central Africa in clade II, South Africa and West Africa in clade III, Middle East and Asia in clade IV, Europe in clade V and Greece in clade VI. The clade IV may be divided into two distinct clades, Asia-1 and Asia-2 [4, 5].

The predominant hosts of CCHFV are wild and domestic mammals and birds [6]. Sheep, goats and cattle develop high titers of virus in blood, but tend to be asymptomatic. Humans are usually infected with CCHFV through a tick bite or close contact with viral-contaminated tissues or blood of domestic animals [6, 7]. Blood and secretion of the infected patients could distribute the virus so medical laboratory staff and healthcare workers are considered as high-risk groups [8, 9].

In 1999, an outbreak was reported from Chahar-mahal and Bakhtiari province, south-west of Iran [10, 11]. According to the latest records, CCHFV exists in 27 of 31 provinces of Iran [5]. The aim of this study was to determine the prevalence of CCHFV infection in hard ticks (Ixodidae) using Reverse Transcription-Polymerase Chain Reaction (RT-PCR) from Chabahar County, Southeast Iran.

## 2 Materials and methods

### 2.1 Study area and sample collection

**Fig. 1:**
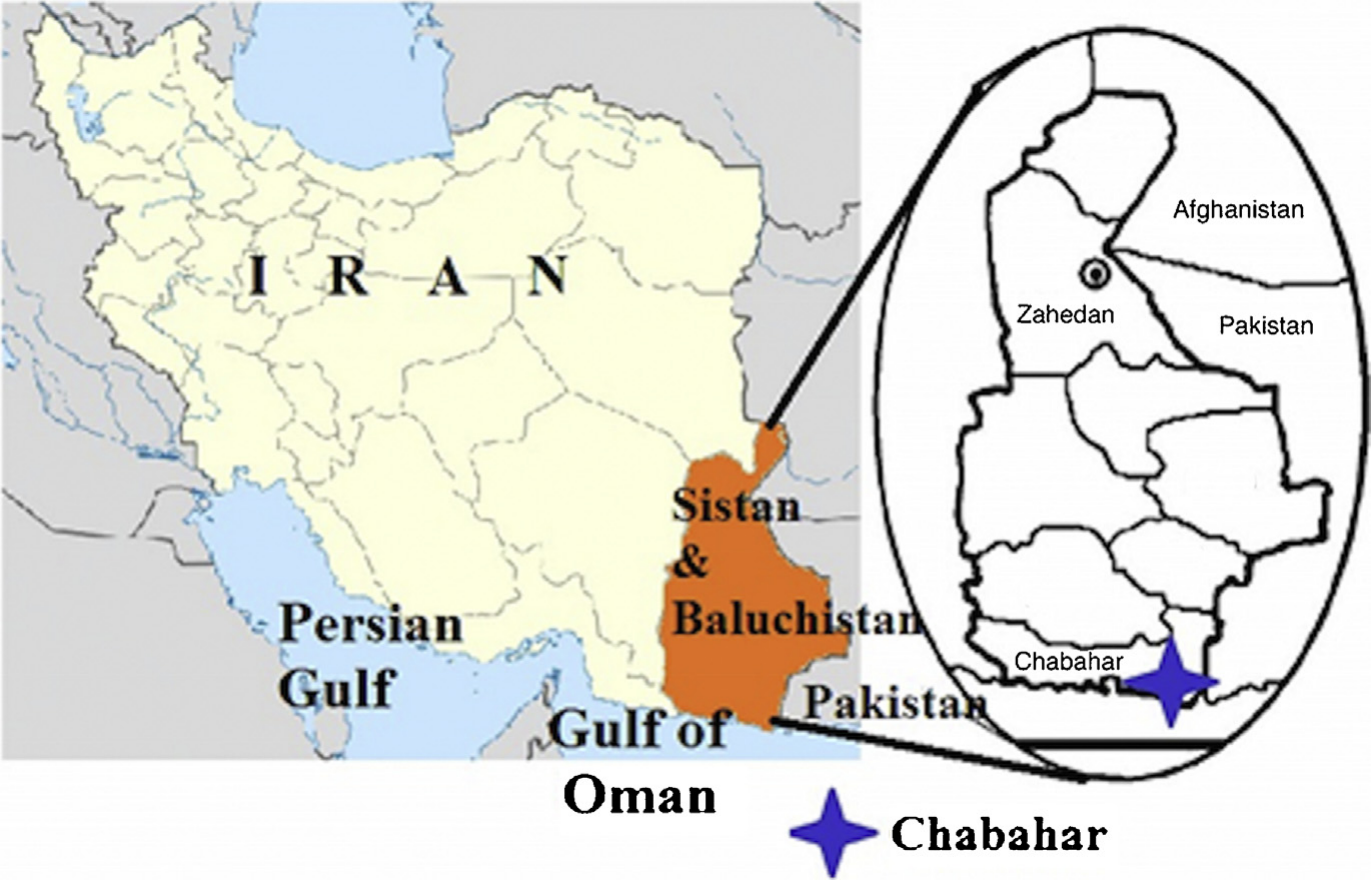
Map of Iran showing our study area. Sistan and Baluchistan Province is located in southeast of Iran. Chabahar County is bordered with Pakistan and Gulf of Oman.

Our survey was carried out in Chabahar County, which is located in Sistan and Baluchistan province, bordering with Pakistan and the Oman Sea. The province is the largest in Iran, subject to seasonal winds from different directions. This province accounts for one of the driest regions of Iran with a slight increase in rainfall from east to west, and an obvious rise in humidity in the coastal regions. At the 2014 census, the county's population was approximately 120,000. The county is subdivided into three districts: the Central District, Dashtiari District, and Polan District. Chabahar county has two cities: Chabahar and Negur (Fig. 1).

During the springtime of 2015, ticks infesting goats, sheep, camels and cows were collected (Fig. 1). In cases of infestation, ticks were collected, ensuring correct personal protective equipment (PPE) was worn and by using forceps to remove the ticks from livestock. All specimens were preserved in tubes and relative information was recorded. Specimens were transferred to the Entomology Laboratory, School of Public Health, Tehran University of Medical Sciences, Tehran, Iran. Ticks were identified to the species level by Janbakhsh and Walker keys [12].

### 2.2 Serological assay

Among 28 livestock included for tick collection, serum samples were obtained from 7 livestock for serology analysis. For IgG detection, Enzyme-linked Immunosorbent Assay (ELISA) plates were coated with mouse hyper immune ascetic fluid and incubated overnight at 4 °C. Diluted recombinant antigen was added to plates. The plates were incubated for 3 h at 37 °C and extensively washed. Serum samples diluted in Phosphate-Buffered Saline containing 0.05% Tween-20 and 5% dry milk (PBSTM) were added, and the plates were incubated for 1 h at 37 °C. After washing, the peroxidase-labeled anti-animal (anti-sheep IgG, anti-bovine IgG or anti-goat IgG) immunoglobulin diluted in PBSTM was added to each well, and the plates were incubated again. After washing, hydrogen peroxide was added, and the plates were incubated at room temperature. The enzymatic reaction was stopped. The plates were read by an ELISA reader at 450 nm [11, 13].

### 2.3 RNA extraction and RT-PCR

Collected ticks were pooled and numbered in 28 tubes. Ticks collected from each host were kept alive in separate labeled tubes, and were then transferred into the laboratory of Medical Entomology, School of Public Health, Tehran University of Medical Sciences, and were identified by morphological characteristic using a stereo- microscope based on valid identification keys of Janbakhsh and Walker [12]. Then, all identified ticks were kept in microtubes and were transferred to the Arboviruses Laboratory, Pasteur Institute of Iran (National Reference Laboratory of Iran) for CCHFV RNA detection by reverse transcription-polymerase chain reaction (RT-PCR) method.

Ticks were individually washed twice with PBS 1X and crushed with a mortar and pestle in 200–300 μl of PBS 1X. Total RNA was extracted from the samples using the RNeasy kit (QIAGEN, Viral RNA mini kit, GmbH, Hilden, Germany) according to the instructions of the supplier. The RNA was dissolved in 50 mL of RNase-free water and stored at −70 °C until use. A master mix was prepared with QIAGEN one step RT-PCR kit (QIAGEN GmbH, Hilden, Germany) and following forward and reverse primers were used (5′TGGACACCTTCACAAACTC-3′) and (5′GACAAATTCCCTACACCA-3′) to amplify a 536-bp inside the S RNA segment. The cycling parameters were as follows: reverse transcription for 30 min at 50 °C, 95 °Cfor 15 min as hot start, 35 cycles of 30 sec of denaturation at 95 ° C, 30 seconds of annealing at 50 ° C, 45 seconds of elongation at 72 ° C with final elongation of 5 minutes at 72 ° C (8, 9). Finally, DNA bands were stained with ethidium bromide and were visualized on a UV transilluminator. Then, positive samples were sequenced according to a previously described method [14].

### 2.4 Phylogenetic analyses

In addition to the CCHFV sequences obtained in this study, 16CCHFV strains retrieved from GenBank at www.ncbi.nih.gov were incorporated into the alignments. The sequence alignment was undertaken using ClustalW and phylogenetic trees were generated by Maximum Likelihood (ML) method with Kimura two-parameter distance using the MEGA-6 software. Bootstrap confidence limits were based on 1,000 replicates [15, 16].

### 2.5 Accession numbers

The nucleotide sequences generated in this study have been deposited in Genbank under accession numbers KU242339- KU242341.

## 3 Results

### 3.1 Serology of livestock

Among 28 livestock, 7 livestock were selected and serum samples were obtained for serology analysis. IgG antibodies against CCHFV were detected in 1 of 7 examined livestock.

### 3.2 Tick species

In total, 21 goats, four cows, two camels and one sheep were examined for the presence of ticks. Ticks were mainly found on the shoulders and ears of livestock. All of the examined livestock were infested with ticks. In total, 49 ticks were collected, all belonging to the genus *Rhipicephalus* and *Hyalomma* (Table 1).

**Table 1:** Details of identified ticks collected from Chabahar district located in Sistan and Baluchistan province, southeast part of Iran.

Code	Host/(F,M)	Genus/species/(F,M)	Collected ticks	RT-PCR (Ticks)	IgG test (Livestock)
1	Goat (M)	*Rh.sanguineus* (M)	1	Neg	**Neg**
2	Goat (M)	*Hy. Spp* (F)	2	Neg	**-**
3	Goat (F)	*Rh.sanguineus* (M)	2	Neg	**-**
4	Goat (F)	*Rh.sanguineus* (F)	2	Neg	**-**
5	Cow (F)	*Hy.marginatum* (F)	1	Pos	**Neg**
6	Goat (F)	*Hy.anatolicum* (M)	1	Neg	**-**
7	Goat (F)	*Hy.anatolicum* (F)	1	Neg	**-**
8	Goat (M)	*Hy.anatolicum* (M)	3	Neg	**-**
9	Goat (F)	*Hy.anatolicum* (F)	2	Neg	**-**
10	Goat (M)	*Hy. spp N*	2	Neg	**-**
11	Goat (F)	*Hy.asiaticum* (M)	1	Neg	**Neg**
12	Goat (M)	*Hy.anatolicum* (M)	1	Neg	**-**
13	Camel (M)	*Hy.dromedarii* (M)	1	Neg	**-**
14	Camel (M)	*Hy.dromedarii* (F)	1	Neg	**-**
15	Goat (F)	*Rh.sanguineus* (F)	2	Neg	**-**
16	Sheep (F)	*Rh.sanguineus* (M)	1	Pos	**Pos**
17	Goat (F)	*Rh.sanguineus* (M)	2	Neg	**-**
18	Goat (F)	*Rh.sanguineus* (F)	2	Neg	**-**
19	Goat (F)	*Rh. spp* (F)	1	Pos	**Neg**
20	Goat (F)	*Hy.anatolicum* (M)	1	Neg	**-**
21	Cow (F)	*Hy.asiaticum* (M)	1	Neg	**-**
22	Cow (F)	*Hy.anatolicum* (M)	4	Neg	**Neg**
23	Goat (M)	*Rh.sanguineus* (M)	4	Neg	**-**
24	Goat (F)	*Rh.sanguineus* (F)	1	Neg	**-**
25	Goat (F)	*Rh.sanguineus* (M)	2	Neg	**-**
26	Goat (F)	*Rh.sanguineus* (F)	2	Neg	**-**
27	Cow (F)	*Hy.anatolicum* (F)	3	Neg	**Neg**
28	Goat (F)	*Rh.sanguineus* (M)	2	Neg	**-**
Total	28 livestock		49	3/49 (6%)	1/7 (14.3%)

M = Male and F = Female.

**Fig. 2:**
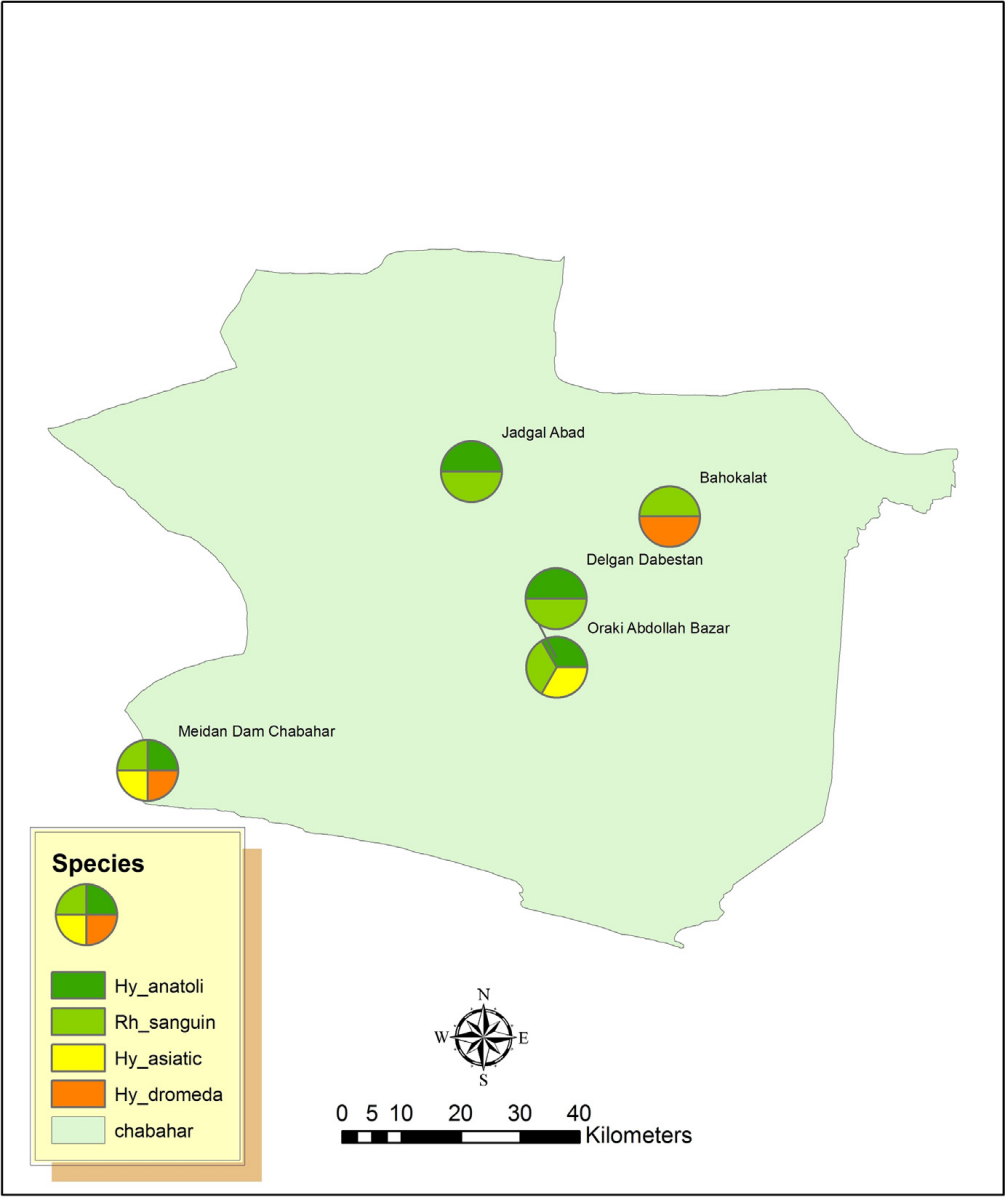
GIS analysis showing the frequency of tick species in different sampling sites in Chabahar County.

Tick prevalence was 49.0% (n = 24) for genus *Rhipicephalus* and 51.0% (n = 25) for genus *Hyalomma*. *Rh. sanguineus* had 46.9% prevalence. Four species of genus *Hyalomma* were indentified as *Hy. anatolicum*, *Hy. asiaticum, Hy. dromedarii* and *Hy. marginatum* with prevalence of 32.7%, 4.1%, 4.1% and 2.1%, respectively. The prevalence of tick species in each sampling sites in Chabahar County are shown in Fig. 2.

### 3.3 CCHFV prevalence in the tick populations

CCHFV RNA detected in three of 49 ticks. Code 5 (IR-T1-HMA-Chabahar-KU242339) obtained from a female *Hy. marginatum*, code 16 (IR-T2-RSA-Chabahar-KU242340) obtained from a male *Rh. sanguineus* and code 19 (IR-T3-RSP-Chabahar-KU242341) obtained from a female *Rhipicephalus spp* collected from a female cow, female sheep and female goat respectively were infected with CCHFV (Table 1).

### 3.4 Phylogenetic analysis of CCHFV strains derived from ticks

**Fig. 3:**
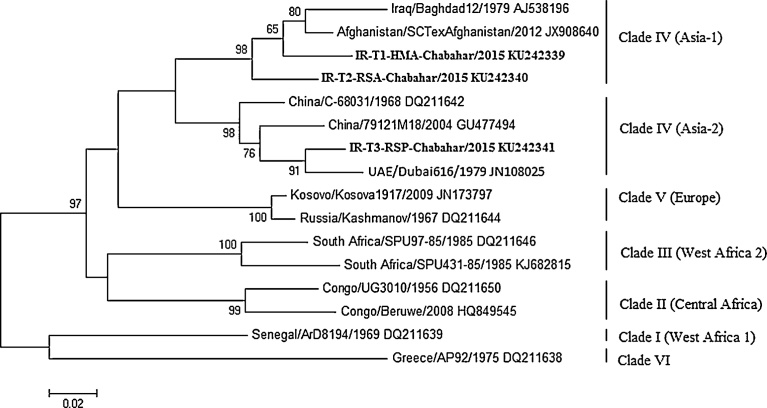
Phylogenetic tree based on the partial sequence of the S-segment of CCHFVs. The tree was conducted by using the ML method with Mega 6 software. The sequences obtained from this study are in bold. The numbers above the branches indicate the bootstrap values in percentages of 1,000 replicates.

Phylogenetic analysis using the partial S-segment demonstrated that 2 sequences (IR-T1-HMA-Chabahar and IR-T2-RSA-Chabahar) clustered in clade IV (Asia-1) with two previously known CCHFV strain from Iraq (Baghdad12) and Afghanistan (SCTexAfghanistan), and 1 sequence (IR-T3-RSP-Chabahar) located within clade IV (Asia-2) with other CCHFV strains China (DQ211642 and GU477494) and UAE (JN108025) (Fig. 3).

## 4 Discussion

Several arboviruses are circulating in Iran [17, 18, 19, 20, 21, 22, 23, 24], but human CCHF cases have the highest number of infections and mortality per annum [25]. In this regards, the southeast of Iran is an endemic area for CCHF. In 1999, some patients with CCHF like-symptoms were reported in the central part of Iran and afterwards, a total of 255 similar cases were recorded in the southeast of Iran including Sistan and Baluchistan province [26]. Recently, a case was reported that was co-infected with CCHFV and malaria in Sistan and Baluchistan province [27].

In terms of serological investigation among suspected human cases for CCHF in Iran between 2000 and 2004, 248 sera were positive for anti-CCHFV antibodies (IgM and IgG). Among 248 confirmed cases, 68% (n = 169) were reported from Sistan and Baluchistan province [28] indicating the high prevalence of CCHFV in this area. In a survey carried out in 2006, three of 300 individuals from the general population were shown to be IgG ELISA positive for CCHFV antibodies and the point estimate of the seroprevalence calculated as 0.024 with 95% confidence interval: 0.003–0.044 [29].

Given CCHFV RNA prevalence among collected ticks, results show that CCHFV RNA is observed in three of 49 ticks (6%). To compare with a previous study conducted in the province, out of 140 collected ticks from livestock, the CCHFV RNA was detected in 4.3% of ticks and the infected tick genera belonged to *Hyalomma* and *Haemaphysalis*. This is also in concordance with another study that 5.3% of collected ticks were positive for CCHFV RNA [30].

However, it was not possible to identify any tick of the genus *Haemaphysalis*, although *Hyalomma* and *Rhipicephalus* ticks infected with CCHFV were identified as part of this study.

Of three molecularly CCHFV positive ticks infesting livestock, one host (sheep) was also serologically positive for IgG antibody against CCHFV, whilst two hosts (cow and goat) were serologically negative for IgG antibody against CCHFV.

These phylogenetic results confirm that the existing isolates can be grouped into seven main clades. This study provides insights into the genetic variability of CCHFV in tick populations from the South-east of Iran. Phylogenetic analysis of CCHFVs in tick populations reveals clustering in clade IV (Asia-1) and clade IV (Asia-2), which is in agreement with previous reports given circulating genomic variants of CCHFV in Iran [16, 31].

An increasing number of CCHFV infections have been reported in people from various regions of Iran. An early seroepidemiological survey in northern/central parts of the country was undertaken in 1974, thus showing the presence of virus in local livestock [32].

Among 298 sheep and 150 goats tested during 2003–2005 in Khorasan province, neighboring Sistan and Baluchistan province, 77.5% and 46% respectively, were shown to be CCHFV IgG positive [33].

It is suggested that on-going surveillance should be undertaken on livestock, high-risk groups of humans, and ticks, to further the understanding of the CCHF disease status in Sistan and Baluchistan provinces.

## Declarations

### Author contribution statement

Ahmad Jafarbekloo: Conceived and designed the experiments; Performed the experiments; Contributed reagents, materials, analysis tools or data.

Nariman Shahhosseini: Conceived and designed the experiments; Performed the experiments; Analyzed and interpreted the data; Contributed reagents, materials, analysis tools or data; Wrote the paper.

Zakkyeh Telmadarraiy: Conceived and designed the experiments; Performed the experiments; Wrote the paper.

Sadegh Chinikar: Conceived and designed the experiments; Wrote the paper.

Ali Haeri: Contributed reagents, materials, analysis tools or data; Wrote the paper.

Norbert Nowotny, Martin H. Groschup: Analyzed and interpreted the data; Wrote the paper.

Anthony R. Fooks: Analyzed and interpreted the data.

Faezeh Faghihi: Performed the experiments; Analyzed and interpreted the data; Contributed reagents, materials, analysis tools or data.

### Funding statement

This work was supported by the Tehran University of Medical Sciences, Tehran, Iran (grant no 23859).

### Competing interest statement

The authors declare no conflict of interest.

### Additional information

Data associated with this study has been deposited at GenBank under the accession numbers KU242339- KU242341.
